# The associations between whole grain, sugar and nutrients intakes in schoolchildren: a cross-sectional study

**DOI:** 10.1186/s40795-023-00807-9

**Published:** 2023-12-08

**Authors:** HC Koo, GP Lim, Satvinder Kaur, KQ Chan

**Affiliations:** 1 Faculty of Applied Sciences, Tunku Abdul Rahman University of Management and Technology, Kuala Lumpur, Malaysia; 2 Faculty of Applied Sciences, UCSI University, Kuala Lumpur, Malaysia; 3 Jeffrey Cheah School of Medicine and Health Sciences, Monash University Malaysia, Selangor, Malaysia

**Keywords:** Schoolchildren, Malaysia, Nutrients, Sugar, Whole grain

## Abstract

**Background:**

Whole grains have gained extensive attention for their contribution to optimal diet quality in the child population. However, little is known about the association between whole grain and sugar intakes. This study aimed to determine whole grain intake and its associations with sugar and other nutrients intakes in schoolchildren.

**Methods:**

A total of 415 healthy Malaysian schoolchildren aged 9–12 years were recruited in this cross-sectional study, through cluster random sampling. Nutrient and sugar intakes were assessed using 3-day 24-hour diet recalls. Whole grain intake was assessed using a validated whole grain food frequency questionnaire.

**Results:**

In these 415 children (9.4–12.7 years), a total of 24 of them have been excluded due to over- and under-reported their dietary intake. Ultimate sample size was 391 children. Overall, consumption of whole grain, fiber, calcium and B vitamins were lower than the recommended intake. However, children consumed protein sufficiently. Whole grain intake was a significant predictor of calorie (β = 0.1011; *p* < 0.001), carbohydrate (β = 0.060; *p* = 0.002), fat (β = 0.107; *p* = 0.044), riboflavin (β = 3.537; *p* = 0.008) and sugar (β = 0.138; *p* = 0.007) intakes, after controlling for sex, age and ethnicity.

**Conclusion:**

The findings provide insight to parents, educators and healthcare professionals in encouraging children to choose whole grain food that is low in sugar and fat. The outcome will also encourage food manufacturing companies to produce healthier whole grain products.

## Introduction

Southeast Asia is a sub-region in Asia, consisting of eleven independent countries, including Malaysia. In recent years, countries in the region experience the double burden of malnutrition challenges: the coexistence of over- and undernutrition [1]. Non-communicable diseases are the leading cause of death in Southeast Asia [2]. Approximately 8.5 million people across the region died from non-communicable chronic diseases in 2019 [3]. On the other hand, several countries in Southeast Asia are regarded as at great risk for micronutrient deficiency [4]. These challenges happened due to the dietary patterns of the populations [5]. Hence, public health strategies and nutrition interventions are desirable to curb the epidemic in Southeast Asia [6].

For centuries, rice and maize have been produced and consumed as the staple cereals in Southeast Asia [7]. Notably, Thailand and Vietnam are major rice exporters in global cereal market [7]. Grains are the dominant source of dietary energy, and substantially contribute to the carbohydrate, protein and essential nutrient intakes in the region [8]. Several countries have recommended the inclusion of whole grains in their dietary guidelines. For example, Malaysian dietary guidelines recommend that at least 50% of the grains should consist of whole grains [9]. Further, The Health Promotion Board from Singapore has recommended for inclusion of sufficient amounts of grains especially whole grains in daily intake [10]. “Pinggang Pinoy” from Philippine recommends to consume more whole grains too [11]. These recommendations are based on decades of increasing scientific evidence regarding the associations of whole grain intake with non-communicable diseases.

In recent years, whole grains have gained considerable attention for their contribution to optimal diet quality among children [12]. Numerous researches have constantly showed the inverse relationships of whole grain intake and the risk of getting several non-communicable diseases and cancers [13]. Substantial research outcomes also demonstrated the positive impacts of whole grain intake on anthropometric measurements in children population [14]. The proposed protective outcome might be attributed to the synergistic properties of the germ and bran components in whole grain [15], as these components are enriched in dietary fiber and several micronutrients, and bioactive compounds [16]. In spite of these health benefits, studies revealed that whole grain intake in Southeast Asia countries still fall well short of the recommended intake [17–19] (48 g/d). Outcome from a nationally representative study in 2015 demonstrated that whole grain was only taken by a small proportion of children in Malaysia (2.3 g/d) [17]. Whole grain intake in Singaporean children population was higher as compared to Malaysian children population (15.3 g/d), but still below the recommended intake, as reported by a national study in 2016 [18]. Further, only a small proportion of Filipino children (one out of twenty) were whole grain consumers as reported in 2013 [19]. Sensory aspects were one of the predominant barriers to whole grain consumption [20]. Ready-to-eat cereals, ready-to-drink cereal beverages and whole grain bread are the greatest contributors of whole grain intake in children population; this scenario is happening in several countries, including Malaysia [17–22]. Since the innovation of whole grain foods should incorporate texture, taste and sensory properties to attract a greater number of children; processed whole grain foods, which may be higher in sugar and fat, easily incorporated into whole grain, have been widely introduced in the market [22].

Malaysia has recorded the highest obesity rate among the Southeast Asian countries, and a Malaysia nationally representative study conducted in 2019 demonstrated an alarming four-fold increase in obesity prevalence among Malaysian population [23]. Hence, the association between whole grain and sugar intakes should be investigated in Malaysia, given that published study has concerned the excessive intake of processed whole grain foods could affect nutrition status of a child [24]. The objective of the present study was to determine the whole grain intake and its association with consumption of sugar and other nutrients in Malaysian children population. The rising prevalence of childhood obesity in Southeast Asia is alarming, and it has been associated with a rising intake of calorie from added sugars [25]. We anticipate that the outcome of the present study will provide insights for food manufacturing companies to pay attention to whole grain product manufacturing and labelling in the region.

## Methods

### Participants recruitment and study design

A total of 415 healthy Malaysian children aged 9–12 years from Kuala Lumpur were recruited for this cross-sectional study through cluster random sampling. Kuala Lumpur is the capital of Malaysia. A full list of 298 national primary schools from the three zones in Kuala Lumpur was obtained from the Education Department of Kuala Lumpur Federal Territory. A total of 3 national primary schools were randomly selected to participate in this study. The present study has received the ethical approval from the Research Ethics Committee of [removed for blind peer review] and was conducted in accordance with the guidelines laid down in the Declaration of Helsinki. Permission to carry out data collection was obtained from the Malaysia Education Ministry and the Education Department from the Kuala Lumpur Federal Territory. Parents/ guardians were provided a comprehensive written information sheet. The present study has obtained the written informed consent from the parents and verbal assent from the children prior to the commencement of the data collection, accordingly.

The Krejcie and Morgan (1970) [26] formulation was used to determine the sample size. Given that Kuala Lumpur has a total of 41,872 schoolchildren aged 9–12 years, with a relative precision of 5%, and a predicted prevalence of 50% for a confidence interval of 95%. The estimated sample size required for the present study was 381 schoolchildren. We added another 10% to the sample taking into consideration the non-response rate, under/ over-reporting the calorie intake, and the loss of information due to incomplete data. The inclusion criteria were: (1) healthy Malaysian children aged 9–12 years and (2) able to understand, write and read the Malaysian national language (Bahasa Malaysia). Children who were absent on the data collection day or anyone with a serious comorbidity requiring treatment were excluded from the study, considering the possibility that this group of children may alter their dietary intake.

### Estimating whole grain intake

We adopted a food frequency questionnaire (FFQ) for whole grain that had undergone validation and reliability testing, and was developed specifically for Malaysian children [27], to assess whole grain intake in the present study. The entire process was completed through a one-to-one interview. Children were first asked if they had consumed the specific whole grain food items from the food frequency questionnaire in the past one week. If they had consumed the whole grain food item, they were then asked how often it was taken and the serving size that was usually consumed at each time. An album compiled of whole grain food pictures and household measurements were used as a guide during the interview. The frequency of whole grain intake from the FFQ was based on the weekly basis; hence, the average daily intake for a particular whole grain food was calculated according to this formula: [frequency of whole grain intake in a week x number of servings at each intake / 7 days]. Following this, the average daily serving size of whole grain food consumed was then multiplied with the whole grain content per serving in the database to estimate the actual daily whole grain intake from each whole grain food consumed. Ultimately, the whole grain content (in gram) for all the whole grain foods consumed by the children were summed up for daily mean whole grain intake.

### Estimating sugar and nutrients intake

Intakes of calorie, sugar and nutrients were assessed using three non-consecutive days of 24-hour diet recalls, consisting of two weekdays and a weekend day over the period of a week, in an one-to-one interview by trained researchers in school, using household measurements, portion sizes and estimated weights of the food consumed. FFQ and 24-hour dietary recall are the most commonly used instruments in epidemiological studies of children [28]; additionally, the 24-hour dietary recall method for estimating nutrient intakes in 10–14 years old children has been validated [29]. Children were requested to recall all the foods and beverages consumed in the past 24-hour. Household items such as spoons, glasses, bowls and dishes in commonly-used sizes, as well as food models and food photo album were used to aid portion sizes estimation during 24-hour dietary recalls. Brand information, ingredients, meal time and cooking method were also recorded where applicable. The average calorie, sugar and nutrients intakes were determined by inputting all the foods and beverages recorded into Nutritionist Pro software (Axxya Systems, United States), based principally on the food product labels and the Nutrient Composition of Malaysian Food. Mean daily calorie, sugar and nutrients intakes over the three days were then calculated.

### Calorie misreporting among children

Calorie intake adopted from the 3-day 24-hour dietary recalls was compared with the estimated basal metabolic rate (BMR) to classify children who might be under-reporting or over-reporting their dietary intake [30]. The standard formula according to age and sex from the FAO/WHO/UNU (1985) [31] was applied to compute the BMR. For a boy, ratio between calorie intake and BMR of less than 1.39 and more than 2.24 be considered under-reporting and over-reporting, respectively [32]. Whereas for girls, ratio between calorie intake and BMR of less than 1.30 and more than 2.20 be considered under-reporting and over-reporting, respectively [32]. Children fell under these categories were excluded from the data analyses that involve dietary data.

### Statistical analyses

Data analyses was conducted using the Statistical Package for the Social Sciences (SPSS) version 22.0 (IBM Corp., Armonk, NY, USA). Data was entered, cleaned and checked before data analysis. The normality of each variable was tested using the Kolmogorov-Smirnov test. Descriptive analysis was conducted for all continuous data. Mean and standard deviation (SD) were presented for normally distributed data; while median and interquartile range (IqR) for data with non-normally distributed. Frequency and percentage were expressed for categorical data. The associations between sociodemographic factors and whole grain intake were determined by Mann-Whitney U test and Kruskal Wallis test. The Spearman’s rank correlation test was conducted to assess the correlations between whole grain intake with sugar and nutrients intakes. General linear regression analyses were performed to study the association between whole grain intake and sugar and nutrient intakes independently of confounding factors. The variables that approached *p* < 0.25 or achieved significance *p* < 0.05 in the simple linear regression analyses were designated as confounders, these including sex, age and ethnicity. A cut off value of 0.25 is supported by literature [33, 34]. Ethnicity was coded as dummy variables whereby Malay was coded as 0 while Chinese, Indian and Others were coded as 1. Assumptions required by the general regression model including linear relationship, multivariate normality, independent errors, outliners, multicollinearity and homoscedasticity were conducted and fulfilled prior to the analyses. In the analyses, dependent variable was whole grain intake; whereas sugar, nutrients intake and all the selected confounders were independent variables. A two-sided *p*-value of < 0.05 was considered statistically significant.

## Results

### Characteristics and calorie misreporting

Table 1 demonstrates the characteristics and calorie misreporting of the children. Of the 415 children (9.4–12.7 years), a total of 16.4% of them did not consume any whole grain. The percentages of boys and girls across whole grain consumer and non-whole grain consumer were similar, although there was a slightly higher proportion of girl in whole grain consumer group. A majority of the children were Malay (n = 277; 66.7%) and had accurately reported their dietary intake (n = 391; 94.2%). Of the 415 children recruited into the study, a total of 24 children were excluded for further dietary intake analyses as they reported their calorie intake inaccurately, our final sample consisted of 391 children for dietary intake analyses.

**Table 1:** Characteristics and calorie misreporting among children (n = 415)

	Total Sample	Whole grain consumer (n = 347)	Non-whole grain consumer (n = 68)
**Age**; median (IqR)	10.8 (1.3)	10.7 (1.3)	10.8 (1.3)
**Sex**; n (%)			
Boys	196 (47.2)	163 (83.2%)	33 (16.8)
Girls	219 (52.8)	184 (84.0%)	35 (16.0)
**Ethnicity**; n (%)			
Malay	277 (66.7)	235 (84.8)	42 (15.2)
Chinese	103 (24.8)	81 (78.6)	22 (21.4)
Indian	34 (8.2)	30 (8.6)	4 (11.8)
Others	1 (0.3)	1 (0.3)	0
**Calorie misreporting;** n (%)			
Under-reporting	24 (5.8)	18 (75)	6 (25)
Normal-reporting	391 (94.2)	329 (84.1)	62 (15.9)
Over-reporting	0	0	0

IqR: Interquartile range

### Overall whole grain, calorie and nutrients intakes

The whole grain, calorie, sugar and nutrient intakes are presented in Table 2. Overall, consumption of whole grain, fiber and calcium were low with the recommended intakes achievement were approximately 26%, 15% and 34%, accordingly. It is remarkable that the levels of calorie, thiamine, riboflavin and niacin were well below the recommended intake too. On the other hand, children on an average had a sufficient intake of protein. Sugar intake was less than 10% of their total calorie intake. Boys tended to consume significantly greater calories (*p* = 0.013), protein (*p* < 0.001) and fat (*p* = 0.008).

**Table 2:** Overall whole grain, calorie and nutrients intakes among the children (n = 391)

Intakes	Sex	Mean	SD	Median	IqR	RNI (%)
Whole grain (g)	Boys (n = 184)	12.5	12.4	9.7	16.6	26.0^¥^
	Girls (n = 207)	12.9	18.5	7.4	15.0	26.7^¥^
Calorie (kcal) ^*^	Boys (n = 184)	1517.7	257.7	1474.6	333.8	89.8
	Girls (n = 207)	1450	218.6	1421.4	284.9	96.7
Carbohydrate (g)^†^	Boys (n = 184)	179.1	41.1	174.3	51.3	-
	Girls (n = 207)	175.2	37.2	173.9	49.7	-
Protein (g) ^**^	Boys (n = 184)	65.0	18.6	64.1	22.2	100
	Girls (n = 207)	57.4	14.5	56.9	17.6	100
Fat (g) ^†*^	Boys (n = 184)	60.3	15.5	58.9	19.2	-
	Girls (n = 207)	57.1	12.2	57.2	17.7	-
Dietary fibre (g)	Boys (n = 184)	3.4	2.1	3.0	2.5	15.0
	Girls (n = 207)	3.5	2.4	2.9	2.6	14.3
Calcium (mg)	Boys (n = 184)	442.9	188.2	404.1	269.6	34.1
	Girls (n = 207)	419.0	188.1	374.3	223.9	32.2
Thiamine (mg)	Boys (n = 184)	0.83	0.51	0.71	0.63	69.2
	Girls (n = 207)	0.89	1.56	0.70	0.70	80.9
Riboflavin (mg)	Boys (n = 184)	0.99	0.55	0.93	0.77	76.2
	Girls (n = 207)	1.03	0.57	0.95	0.75	100
Niacin (mg)	Boys (n = 184)	10.36	5.46	9.99	7.86	64.8
	Girls (n = 207)	10.11	5.70	9.13	6.55	63.2
Sugar (g)	Boys (n = 184)	22.4	14.2	20.9	19.3	5.9^‡‡^
	Girls (n = 207)	22.4	15.4	20.5	21.9	6.2^††^

RNI: Recommended Nutrient Intakes for Malaysia 2017; IqR: Interquartile range

^¥^ Based on the USDA recommendation of 48 g per day

^‡‡^Based on the World Health Organization recommendation, sugar intake should be less than 10% of total energy intake

Mann-Whitney U test applied; ^†^ Independent sample t-test applied; ^*^p < 0.05; ^**^p < 0.005

### General linear regression for calorie, nutrients and sugar intakes predicted by whole grain intake

As demonstrated in Table 3, whole grain intake was a significant predictor of calorie, carbohydrate, fat, riboflavin and sugar intakes, after controlling for sex, age and ethnicity. It is noticeable that calorie intake increased by 0.011 SD for every one SD increased in whole grain intake. A one SD increased in whole grain intake led to 0.060 SD increased in carbohydrate intake. As for fat intake, one SD increased in whole grain intake would increase fat intake by 0.107 SD. Meanwhile, one SD increment in whole grain intake led to 3.537 increase in riboflavin intake. Our findings also showed that a one SD increase in whole grain intake will lead to a 0.138 SD increase in sugar intake. In summary, whole grain intake increased the calorie, carbohydrate, fat, riboflavin and sugar intakes in the children.

**Table 3:** Summary of general linear regression for whole grain intake predicted by calorie, nutrients and sugar intakes

	Whole grain	
	b (95% CI)	*p*-value
Calorie	0.011 (0.005,0.017)	< 0.001^***^
Carbohydrate	0.060 (0.022, 0.097)	0.002^**^
Protein	0.052 (-0.035, 0.140)	0.240
Fat	0.107 (0.003, 0.211)	0.044^*^
Dietary fibre	0.452 (-0.267, 1.171)	0.217
Calcium	0.006 (-0.001, 0.014)	0.107
Thiamine	0.717 (-0.570, 2.003)	0.274
Riboflavin	3.537 (0.911, 6.163)	0.008^**^
Niacin	0.264 (-0.001, 0.530)	0.051
Sugar	0.138 (0.038, 0.238)	0.007^**^

Adjusted regression coefficient for age, sex and ethnicity

General linear regression method applied. Models assumptions were fulfilled. There were no interactions among independent variables. No multicollinearity detected. Ethnicity was coded as dummy variables whereby Malay was coded as 0, while Chinese, Indian and others ethnicity were coded as 1. ^*^p < 0.05; ^**^p < 0.01; ^***^p < 0.001

## Discussion

The present study aimed to determine the associations between the intakes of whole grain and sugar, as well as other nutrients intakes in Malaysian children’s population. To our knowledge, this is the first cross-sectional study in Southeast Asia region to determine the association between whole grain and sugar intakes. The present study delivers novel data and reveals positive associations between whole grain, sugar and several nutrient intakes. Considering the growing rate of childhood obesity prevalence in Southeast Asia, which is associated with the increasing sugar intake in the region [25], the outcomes of the present study may provide insight to parents, educators and healthcare professionals in encouraging children to select whole grain foods that are low in sugar and fat. It will also remind food manufacturing companies to produce healthier whole grain products.

It is worth nothing that the proportion of whole grain consumers among the schoolchildren in the present study (83.6%) has improved as compared to the previously reported data (55.6%) from a similar background population in Kuala Lumpur [16]. This improvement might be attributed to several whole grain promotion campaigns and interventions in the country. For instance, the GReat-Child Trial aimed to improve the availability and awareness of whole grain intake among schoolchildren in Kuala Lumpur [35]. The Nutrition Society in Malaysia also organized several whole grain promotion campaigns among the population [17]. Nonetheless, habitual intake of whole grains has improved but still falls well short of the recommended intake (48 g/day) [36]. This finding is comparable to published data from other regions in Southeast Asia, including Singapore and the Philippine. The intake of whole grain in Singaporean children (15.9 g/d) was similar to our present study [18], whereas only a small proportion of Filipino children (one in twenty) were whole grain consumers [19]. The present study did not investigate the barriers to whole grain intake among the children; however, several studies have demonstrated that sensory and food preparation skills are the biggest barriers to whole grain intake in the Asian population [20, 37]. A qualitative study from Malaysia has suggested that a whole grain cookbook specifically for the Asian population may improve whole grain intake in the region [38]. Perhaps, whole grain snack items (granola bars and oatmeal cookies) may have greater effect in improving whole grain consumption among children compared to whole grain staple food items (brown rice and oatmeal porridge); however, those items tend to contain higher sugar and fat, which may negate the health benefits that higher whole grain intake could confer [37].

Besides whole grain, children in the present study demonstrated low intake of fiber, calcium and B vitamins too. It is in accordance with studies from other countries in the region, including Singapore [39], Indonesia [40] and Vietnam [41]. Nutrient deficiency is not only a persistent issue across Southeast Asia, it is also a global health issue in the present-day, hindering children’s physical, cognitive and psychosocial developments [42]. Vitamins and minerals are the key drivers in health maintenance and disease prevention in a child, it is rather important to achieve rapid linear pubertal growth in children aged 9–12 years [43]. Food-based approaches such as food fortification and improving whole grain intake are the main strategies to improve intakes of vitamins and minerals among children [44]. Children are encouraged to have a diversified diet in all meals to ensure an adequate intake of essential nutrients [45].

Over the past few years, whole grain intake and its associations with other nutrient intakes have drown much attention, particularly in children’s diet. Findings of our study have indicated that higher intake of whole grain was significantly associated with greater intake of B vitamins. Our finding may reflect the naturally higher content of B vitamins in whole grain foods, which are lost in refined grain during processing. However, it is challenging to exclude any potential confounding from the overall healthy lifestyle and diets, which come hand in hand with whole grain intake; hence, it is uncertain whether these significant associations in B vitamins are completely due to the higher whole grain consumption or the combination effects of an overall healthy diet. Nonetheless, higher intake in whole grain improved B vitamins has also been demonstrated in several published journals [22, 46] and associated with greater overall diet quality in children’s population [17].

The present study demonstrated that higher whole grain intake may increase sugar and fat intakes in children. These findings should be viewed with caution, given that a majority of our respondents consumed processed whole grain foods that contain higher amount of sugar and fat [37] such as ready-to-eat cereals and biscuits. It is similar to a number of countries including Singapore [37] and Australia [21], where ready-to-eat cereals, bread and biscuits are the main contributors to whole grain intake. Emphasizing sufficient whole grain intake may be a better strategy to assist parents in incorporating healthy foods into their children’s diet and lead to a higher intake of fiber, calcium and B vitamins. However, parents should choose whole grain products wisely and read the ingredient list carefully. Perhaps, whole grain promotion campaigns should rather emphasize whole grain staple items and avoiding processed whole grain items that are high in sugar and fat.

To the best of our knowledge, this was the first study in Southeast Asia to investigate the association between whole grain intake and sugar, as well as other nutrient intakes. Most of the food items were analyzed at the brand level, especially whole grain products. We directly contacted the manufacturers to obtain the information in case the food label or NutriPro Software did not clearly indicate the product contents. Besides, we have adjusted for several potential confounders and also excluded the children who had under-overreported their calorie intake; it is a useful method to account for the plausibility of self-reported calorie intake in a human study [47].

The limitation that arise from the cross-sectional study design must be considered; hence, statements about the causality of associations are impossible. Besides, self-reported dietary assessment in children is challenging, as children may not be able to describe dietary intake in detail or might introduce social desirability bias. To minimize this limitation, confidentiality of individual results had been reassured. We have also excluded those children who had over/underreported their calorie intake. Perhaps, future researchers may adopt nutritional biomarkers, wearable devices, or image-assisted dietary assessment methods to improve the accuracy of dietary intake estimation.

On the whole, findings of the present study indicate that whole grain intake has significant positive associations with B vitamins, sugar and fat intakes, after controlling for sex, age and ethnicity. In this paper, we provide insight to food manufacturing companies to reformulate the whole grain products with priorities towards health-centric consumer preferences with low-sugar and fat content. It may also contribute to strengthening of public policies aiming to improve food labeling constitution in Southeast Asia, particularly in Malaysia.

## Data Availability

The data used and/ or analyzed during the current study are available from the corresponding author on reasonable request.
